# IgG4-Related Disease With Tuberculosis: A Case Report and Retrospective Review of Patients in a Single Center

**DOI:** 10.3389/fimmu.2021.652985

**Published:** 2021-04-21

**Authors:** Pingying Qing, Chenyang Lu, Zhihui Liu, Xiuzhen Wen, Bo Chen, Zhiguo Lin, Yingbing Ma, Yi Zhao, Yi Liu, Chunyu Tan

**Affiliations:** ^1^Department of Rheumatology and Immunology, West China Hospital, Sichuan University, Chengdu, China; ^2^Laboratory of Rheumatology and Immunology, West China Hospital, Sichuan University, Chengdu, China; ^3^Department of Rheumatology and Immunology, Jiujiang No.1 People’s Hospital, Jiujiang, China; ^4^Department of Rheumatology and Integrated TCM & Western Medicine, Baiyin Second People’s Hospital of Gansu Province, Baiyin, China; ^5^Department of Rheumatology and Endocrinology, Kaiyuan People’s Hospital, Kaiyuan, China

**Keywords:** IgG4-related disease, tuberculosis, intracranial, IFN-gamma release assay, retrospective cohort

## Abstract

**Background:**

IgG4-related disease (IgG4-RD) is a recently recognized systemic fibro-inflammatory disease of unknown cause involving many organs including pancreas, salivary glands, and lymph nodes. Chronic tuberculosis (TB) infection has been reported in IgG4-RD, but the prevalence of TB infection has not been evaluated in IgG4-RD.

**Methods:**

Characterization of a patient with IgG4-RD by physical examination, laboratory tests, magnetic resonance imaging (MRI) and histological examination. TB infection was evaluated by medical history, radiological examinations, sputum examinations, tubercular skin test (TST) and interferon gamma (IFN-γ) release assay test (IGRA). Medical records of IgG4-RD patients were reviewed in our institute from February 2015 to September 2020 to explore the prevalence of TB infection in IgG4-RD.

**Results:**

We described a 40-year-old Chinese man presented with headache and diplopia. Physical examination revealed bitemporal hemianopsia and limited abduction of both eyes. MRI revealed uniformly enhancing mass overlying clivus with dural tail sign. Laboratory data revealed elevation of IgG4 (1.9g/L), and TB-IGRA demonstrated significantly elevated IFN-γ (414.21 pg/ml). The clivus lesion was subtotally removed and IgG4 was strongly positive on immunohistochemical staining. The diagnosis of IgG4-RD was established, and the patient received treatment of corticosteroids, methotrexate, and cyclophosphamide with isoniazid prophylaxis. Consequently, the mass shrank remarkably within 3 months. A similar concurrence of TB disease or latent TB infection (LTBI) and IgG4-RD was present in 17/47 (36.2%) patients in our institute.

**Conclusion:**

High frequency of TB/LTBI presented in patients with IgG4-RD. Patients with IgG4-RD and LTBI should be closely monitored for resurgence of TB. Whether TB represents a risk for IgG4-RD should be further investigated in prospective cohort.

## Introduction

IgG4-related disease (IgG4-RD) is a novel syndrome that forms an inflammatory pseudotumor with increasing IgG4 positive plasma cells and lymphocytes infiltrating tissues, such as lacrimal gland, salivary gland, pancreas, bile duct, kidney, and retroperitoneum (1). Histologically, irrespective to disease site, it is characterized by densely infiltrated IgG4 positive plasmacytes along with storiform fibrosis and obliterative phlebitis (1, 2). Along with increased recognition of IgG4-RD, increasing number of patients are diagnosed with IgG4-RD. However, the pathogenesis of IgG4-RD remains largely unknown.

Previous reports described that IgG4-RD may be associated with presentation of autoantigen by plasmablasts or B cells to CD4+ cytotoxic T cells, causing cell death and fibrosis (3). However, little is known about the antigens instigating the disease. Autoimmune pancreatitis, of which type I is now considered IgG4-related pancreas disease, is related to *Helicobacter pylori* infection (4). Moreover, reports of co-concurrent *Mycobaterium tuberculosis* (*M. tb*) infection in IgG4-RD have been reported consecutively (5–11). Complication of tuberculosis (TB) in autoimmune disease usually calling attention to the association of TB with immunosuppressive treatments such as steroids, conventional disease modifying anti-rheumatic drugs (cDMARDs) and bioagents. However, some patients suffer from TB before development of IgG4-RD (5–8). Whether *M. tb* infection is a risk factor or complication of IgG4-RD is unclear.

Individuals infected with *M. tb* may develop symptoms (TB disease) or may have no clinical evidence of disease (latent TB infection, LTBI) (12). LTBI is a special state of persistent bacterial viability, immune constraint and no evidence of clinically manifested active TB (13). The diagnosis of TB or LTBI is mainly based on medical history, radiological examinations, sputum examinations, tuberculin skin test (TST) and interferon-γ release assays (IGRA). TST is based on a type IV hypersensitivity skin reaction to tuberculin, a protein derived from *M. tb*. However, this test has low specificity and may have false positives in several conditions such as BCG vaccination or non-tuberculous bacterial infection (14). IGRA rely on the fact that memory T cells generated after TB exposure release interferon-γ(IFN-γ) upon re-exposure to TB antigens. Although has a higher specificity in LTBI diagnosis, IGRA present some economic and organizational constrains. Lacking a gold standard method for the diagnosis of LTBI, TST and TB-IGRA remain the primary screening tools despite having major limitations (14).

Here we reported a case of IgG4-related clivus lesion in a patient with LTBI. The co-occurrence of IgG4-RD and TB infection in the same patient prompted us to explore whether this phenomenon is frequent in a retrospective IgG4-RD cohort.

## Results

### IgG4-Related Intracranial Disease in a Patient With Latent Tuberculosis Infection

**Figure 1:**
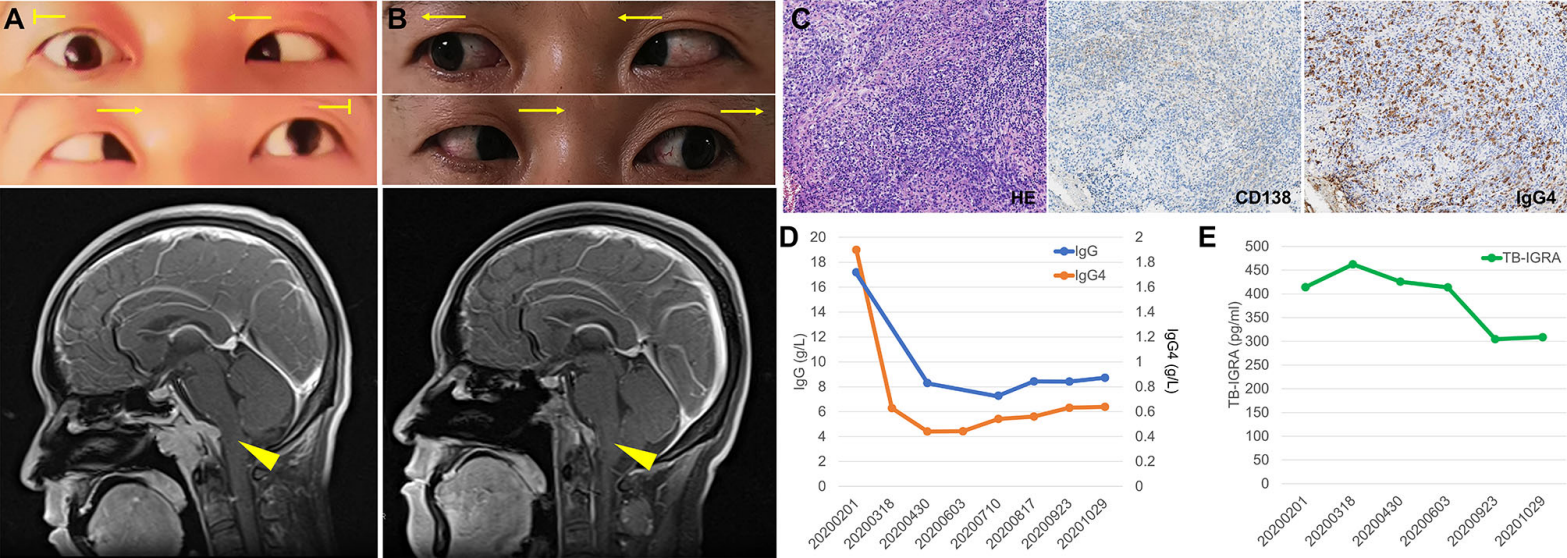
Imaging, histologic and laboratory findings of the case. **(A)** The abduction of both eyes was limited (arrows indicate the direction of movement), and contrast-enhancing brain magnetic resonance imaging (MRI) showed a 3.8×2.9×2.9 cm mass overlying clivus with dural tail sign (arrow head). **(B)** Abduction of both eyes recovered after treatment and MRI showed the mass in the clivus area was remarkably smaller. **(C)** Histologic features of the lesion revealed fibro-connective tissue with mixed inflammation containing predominantly plasma cells and immunochemical analysis revealed an increased number of IgG4-positive plasma cells (×200). **(D, E)** Serum levels of IgG and IgG4 **(D)** and T cell-released INF-γ in TB-IGRA **(E)** of the patient during the follow-up.

A 40-year-old man presented with intermittent headache and diplopia. 6 years before admission, he had recurrent headache at top and frontal area. He had been diagnosed with tubercular meningitis in the local hospital and received HRZE regimen for 2 months followed by HRE regimen for 27 months. His headache was not alleviated and then he went to our hospital 3 years ago. Contrast-enhanced magnetic resonance imaging (MRI) demonstrated an enhancing mass overlying clivus with dural tail sign. The lesion was initially considered to be a meningioma based on its imaging appearance. The patient then received a gamma knife surgery. The headache mitigated a little but persisted to date. 7 months before returning to our institution, he developed diplopia, hoarseness, and dysphagia progressively. On examination, the patient had bitemporal hemianopsia and limited abduction of both eyes (**Figure 1A**, upper). He had no speech disturbance or motor dysfunction. Contrast-enhancing MRI showed a 3.8×2.9×2.9 cm mass overlying clivus with dural tail sign (**Figure 1A**, lower). The lesion oppresses brain stem, with bone erosion at the clivus and left talus cone region and part of the lesion protrudes into pharyngonasal cavity. Cerebrospinal fluid examination was generally normal and mycobacterium spp. and cryptococcus were not detected. He received a surgery with endoscope removing mass subtotally. The histopathological evaluation revealed fibro-connective tissue with mixed inflammation containing predominantly plasma cells. Immunostaining showed increased number of IgG4-positive plasma cells (>200/HPF) and elevated ratio of IgG4-positive cells to CD138-positive cells (approximately 55%, **Figure 1C**). No granuloma with caseous necrosis was noted. These findings were suggestive of an IgG4-RD and the patient was transferred to our department.

Immunoglobulin analysis revealed IgG 17.2 g/L (normal range 8.0-15.5 g/L), IgG4 subclass 1.9 g/L (normal range 0.035~1.5 g/L). Serum levels of IgA, IgM, C3, and C4 were normal. Antinuclear antibody revealed 1:100 titer, while anti-neutrophil cytoplasmic antibodies, anti-SS-A, anti-SS-B, anti-dsDNA, anti-Smith, anti-Scl-70 as well as other anti-ENA were all negative. HCV antibody, HBV surface antigen and HBV-DNA were negative. TST was positive (induration ≥10mm) and TB-IGRA demonstrated significantly elevated IFN-γ (414.21 pg/ml, normal range 0-14 pg/ml). Repeated sputum tests were negative. A computed tomography scan of her chest showed neither lung lesions suggestive of TB nor hilar or mediastinal lymph node swelling. Investigation of other systemic manifestation of IgG4-RD was negative. We diagnosed the patient with IgG4-RD complicated with LTBI. Treatment of corticosteroids combined with methotrexate (10mg/week) and cyclophosphamide (0.5g/m^2^/month for 3 months) was initiated and was well tolerated by the patient. Isoniazid was also started to prevent TB recurrence. In a follow-up of 3 months, he had attenuated bitemporal hemianopsia and better abduction of both eyes, and MRI showed the clivus mass shrank remarkably (**Figure 1B**). Serum IgG consecutively decreased to 8g/L and became stable, while IgG4 level decreased to between 0.4-0.6 g/L along 9 months follow-up (**Figure 1D**). In addition, the patient did not complain any TB related syndromes and the IFN-γ level in the TB-IGRA test decreased gradually (**Figure 1E**).

### Literature Review on Co-Existence of Tuberculosis Infection and IgG4-RD

The cause of IgG4-RD remains unclear, however, chronic infection such as TB was reported in IgG4-RD (summarized in **Table 1**). Notably, IgG4-RD occurs either after TB contact or diagnosis, or at the same time as TB confirmation. Among those patients, TB occurs in lung, urinary tract, lymph node and meninges.

**Table 1:** Case review of IgG4-related disease associated with tuberculosis.

Author, year	Age, sex	Findings	Organ involvement	TB examination	History of TB infection	Time of TB diagnosis
Narang (5)	64, M	Paravertebral and retroperitoneal soft tissue thickening	Retroperitoneal area	NA	TB contact	8 years before
Colombier (9)	61, F	Enlarged ascending aorta with a thickened wall	Aorta	Positive enzyme-linked immunospot assay	None	meanwhile
Bajema (6)	68, M	Bilateral lobe fibrosis, left apical masses, and submandibular mass	Lungs, submandibular glands	*M. tb* was isolated from tissue culture	Pulmonary TB	50 years before
Erlij (10)	40, M	Pericarditis, lymphadenopathy and aortitis	Pericardium, lymph nodes, aorta	Positive TST test and positive Quantiferon test, but negative baciloscopies	None	meanwhile
Imai (7)	63, F	Swelling in submandibular glands, pancreas, and right kidney	Kidney, submandibular glands, pancreas	Negative PCR, positive QuantiFERON-TB test	Urinary tract TB	5 years before
Suzuki (11)	68, M	Sialadenitis, interstitial penumonitis	Salivary glands and lungs	*M. tb* was cultured from the pleural effusion; histologic examination showed epithelioid granuloma	None	meanwhile
Kawano (8)	64, F	Swelling in right lacrimal gland, submandibular glands and lymph nodes	Lacrimal glands, submandibular glands and lymph nodes	Lymph node histology: epitheloid granuloma with multinucleated giant cells and caseation necrosis. Positive TST and QuantiFERON-TB test, but smears, cultures and PCR were negative	Tuberculous lymphadenitis	3 years before

TB, tuberculosis; TST, tuberculosis skin test; M. tb, mycobacterium tuberculosis; PCR, polymerase chain reaction; NA, not available.

### Occurrence of Tuberculosis Infection in Patients With IgG4-RD

China has always been one of the countries with the most serious TB epidemic all over the world. In 2017, about 60.08/100’000 cases of TB were reported in China (15). In addition, skin test positivity ranged from 15% to 42%, and IGRA positivity rate ranged from 13% to 20% in rural China (16). TB is closely related to patients with rheumatic diseases who are receiving immunosuppressive therapies, however, TB incidence in IgG4-RD patients remains unknown. We performed an additional retrospective study to explore the frequency of TB infection in IgG4-RD patients at our institute (**Supplementary Figure S1**). According to the 2011 comprehensive diagnostic criteria (17), we identified 83 subjects with a diagnosis of IgG4-RD who visited our hospital between February 2015 and September 2020. Among them, 47 were screened for TB infection. In 17/47 (36.2%) patient with IgG4-RD, a TB disease (positive signs of TB disease symptoms and chest computed tomography and positive TB tests) or LTBI diagnosis (negative signs of TB diseases and chest computed tomography (CT), *M. tb* was not isolated from a clinical specimen, but positive TB-IGRA and/or TST) (12, 18) was present (**Supplementary Figure S1**). Among the 17 subjects, 82.4% were male and the median age is 52.5 ± 14.3 years. Except two patients (No. 4 and 7), all other patients had high levels of IgG4 in the serum. Two patients (No. 2 and 15) had TB disease based on positive TB symptoms, chest CT, and TB blood test/TST, while others had LTBI. Organ involvement, laboratory examinations, and diagnosis in these patients are summarized in **Table 2**.

**Table 2:** Characteristics of IgG4-related disease with tuberculosis infection in our cohort (2015-2020).

Patient No.	Age, sex	Organ involvement	Serum IgG4 (g/L)	Tissue biopsy	Diagnosis of IgG4-RD	TB screen	Diagnosis of TB
1	51, M	Lung	3.43	+	definite	TB-IGRA (T-N) 389.71 pg/ml; TST +; a history of tuberculous lymphadenitis	LTBI
2	78, M	Retroperitoneal fibrosis	17.00	NA	possible	Night sweets, positive chest CT, TB-IGRA (T-N) 284.75 pg/ml, Xpert MTB/RIF +	TB
3	54, M	Lymph node	3.35	+	definite	TB-IGRA (T-N) 60.2 pg/ml; TST +	LTBI
4	61, M	Pancreas	0.144	+	probable	TB-IGRA (T-N) 19.49 pg/ml; TST -	LTBI
5	51, M	Pancreas, biliary tract	12.40	+	definite	TB-IGRA (T-N) 23.88 pg/ml; TST +	LTBI
6	65, M	Retroperitoneal and periorbital soft tissue, lymph node	7.14	–	possible	TB-IGRA (T-N) 145.58 pg/ml; TST +	LTBI
7	52, M	Salivary gland	0.177	+	probable	TB-IGRA (T-N) 265.72 pg/ml; TST +	LTBI
8	67, M	Liver	2.15	+	definite	TB-IGRA (T-N) 433.61 pg/ml; TST +	LTBI
9	37, M	Periorbital soft tissue, lymph node	10.70	+	definite	TB-IGRA (T-N) 42.34 pg/ml	LTBI
10	59, M	Periorbital soft tissue	14.20	+	definite	TB-IGRA (T-N) 90.27 pg/ml	TLBI
11	40, M	Intracranial	1.90	+	definite	TB-IGRA (T-N) 413.7 pg/ml, a history of tubercular meningitis	LTBI
12	26, F	Periorbital soft tissue	2.13	+	definite	TB-IGRA (T-N) 397.82 pg/ml; TST +	LTBI
13	62, M	Retroperitoneal soft tissue	27.80	+	definite	TB-IGRA (T-N) 228.92 pg/ml; TST +	LTBI
14	46, F	Lacrimal glands and salivary glands	8.84	+	definite	TST +; TB-IGRA (T-N) -	LTBI
15	48, M	Lacrimal glands, pancreas	15.10	+	definite	Cough and night sweets, positive chest CT, TB-IGRA (T-N) 349.78 pg/ml; TST +	TB
16	27, F	Cricoid cartilage, pancreas	2.72	+	definite	TB-IGRA (T-N) 54.56 pg/ml	LTBI
17	68, M	Lacrimal glands, salivary glands, pancreas, lymph node, prostate	23.10	+	definite	TB-IGRA (T-N) 37.48 pg/ml	LTBI

M, male; F, female; TB, tuberculosis; TST, tuberculosis skin test, induration ≥5 mm was considered positive; IGRA (T-N): interferon-gamma release assays for tuberculosis, (test minus nil control, normal range 0-14 pg/ml); tissue biopsy +: ratio of IgG4+/IgG+ or CD138+ plasma cell >40% and >10 IgG4+ plasma cell/HPF. The diagnosis of IgG4-RD was made according to the 2011 comprehensive criteria for IgG4-RD.

## Discussion

Patients with rheumatic diseases are vulnerable to infections because of the intrinsic immune dysregulation associated with the disease processes, the immunosuppressive therapies used, and other associated comorbidities. T-SPOT.TB-positive rates in patients with rheumatic diseases were higher than healthy controls and an 11.2% positive conversion rate was present in those receiving biologics (19). Per our cohort, IgG4-RD patients have a higher prevalence of TB infection, either before the development of autoimmune condition or occurs simultaneously.

IgG4-RD is considered a biphasic progression featured by an “inflammatory” phase and a “fibrotic” phase (20). Various immune cells including plasmablasts, effector memory T cells, cytotoxic T cells, follicular T helper cells, and abundant pro-fibrotic molecules such as IFN-γ, IL-1β, IL-6, and transforming growth factor β were found at the disease sites (20). Clonal expansion of the pathogenic immune cells in patients’ tissues and peripheral blood indicates that IgG4-RD is likely sustained by an antigen driven immune response. A variety of self-antigens have been identified, including galectin-3, annexin-A11, laminin-511, and prohibitin, suggesting that a breach of immunological tolerance might initiate the disease (20). However, the nature of antigens initiating IgG4-RD and how do they alter immune tolerance at the disease sites are largely unknown. Chronic infection such as *M. tb* infection had been reported in IgG4-RD (summarized in **Table 1**). Our present case adds to the literature linking IgG4-RD with TB infection. Notably, IgG4-RD occurs either after TB contact or diagnosis, or at the same time as TB confirmation. Since TB typically has granulomatous inflammation including formation of multinucleated giant cell formation giant cell and granuloma, which is one of the exclusion criteria for IgG4-RD (21), careful pathological examination should be performed for the differential diagnosis.

Diverse autoimmune phenomena were found in TB. For instance, antibodies against citrullinated peptide can be found in almost 40% of TB patients (22). Polyarthritis can be found in TB patients in the absence of detectable bacteria in lesions (23). Given that autoimmunity is involved in the pathogenesis of TB, TB was now considered to be an infection-initiated autoimmune disease (24). Activation of T helper (Th) 1 cells and expression of related cytokines such as IFN-γ are critical in defense of *M. tb (*25). *M. tb* also promotes Th2 immune responses by altering the balance of T cell polarizing cytokines such as IL-1β in infected cells (26). Moreover, elevated serum IgE can be found in TB patients and treatment against TB reduces IgE concentrations (27). Intriguingly, abundant IFN-γ-secreting cytotoxic CD4+ T cells were found to be involved in the lesional damage of IgG4-RD (28). IgG4-RD are often complicated with allergic diseases (29), meanwhile, Th2 cells are usually activated in these patients and secrete cytokines which could promote class switching to IgG4 and IgE (30). Therefore, IgG4-RD share some common immune responses with TB infection. This case study highlights that TB could be a possible pathogen linking IgG4-RD through a hypersensitive mechanism. However, further analysis of longitudinal case-control cohorts and basic experiments are warranted to clarify the role of TB in the development of IgG4-RD.

IgG4-RD is highly treatable with either glucocorticoids or targeted biological drugs like B cell-depletion agents (1, 3). Glucocorticoids are usually the first-line therapy for IgG4-RD when diagnosis is confirmed (31). However, glucocorticoids often fail to induce durable remissions and complications from lengthy courses of high-dose steroids is substantial. Increasing data suggest that a B cell-depletive bioagent rituximab as well as some cDMARDs such as cyclophosphamide, azathioprine, mycophenolate mofetil, methotrexate, tacrolimus and leflunomide offer additional benefits in the management of IgG4-RD (32–34). Moreover, many studies have shown combination therapy such as cell cycle-specific agent methotrexate and cell cycle-nonspecific agent cyclophosphamide is well tolerated and have synergistic anti-inflammatory properties (35, 36). But all those immunosuppressive agents would inevitably increase opportunistic infection risks, especially when IgG4-RD was complicated with TB. In our case, the patient had recurrent mass in the narrow area, and surgery only removed the mass partially to avoid damaging adjacent vital tissues. Considering the recurrence nature of the disease, unsatisfactory surgical results and economic condition of the patient, prednisone combined with two cDMARDs with different mechanisms were administrated. The patient achieved infection-free remission with glucocorticoids and cDMARDs combined with preventative antituberculous therapy. However, randomized clinical trials of therapies against IgG4-RD, especially those concurrent with infection diseases including TB, needs to be launched and long-term follow-up still lacks.

## Data Availability Statement

The original contributions presented in the study are included in the article/**Supplementary Material**. Further inquiries can be directed to the corresponding author.

## Ethics Statement

The studies involving human participants were reviewed and approved by Sichuan University West China Hospital Health Research Ethics. The patients/participants provided their written informed consent to participate in this study. Written informed consent was obtained from the individual(s) for the publication of any potentially identifiable images or data included in this article.

## Author Contributions

PQ, ZLin, and YM: case report data collection and analysis, writing of the original draft. ZLiu, XW, and BC: retrospective search for IgG4-RD patients and data analysis, manuscript review/editing. CL: methodology for the database search, data collection, and manuscript review/editing. YZ, YL, and CT: conceptualization, methodology, data visualization, writing of the original draft, and supervision. PQ and CL have contributed equally to this article. All authors contributed to the article and approved the submitted version.

## Funding

This study was supported by the National Natural Science Foundation of China (No. 81501412) and the 1·3·5 project for disciplines of excellence, West China Hospital, Sichuan University (Grant No: ZYGD18015, ZYJC18003).

## Conflict of Interest

The authors declare that the research was conducted in the absence of any commercial or financial relationships that could be construed as a potential conflict of interest.
