# Supplementary material for: IgG4-Related Disease With Tuberculosis: A Case Report and Retrospective Review of Patients in a Single Center
Source: Front Immunol. 2021 Apr 21;12:652985. doi: 10.3389/fimmu.2021.652985 (PMC8097037; doi:10.3389/fimmu.2021.652985)
Supplement: Supplementary file 1 [file DataSheet_1.docx]

**Supplementary material for**

**IgG4-related disease with tuberculosis: a case report and retrospective review of patients in a single center**

Pingying Qing §, Chenyang Lu §, Zhihui Liu, Xiuzhen Wen, Bo Chen, Zhiguo Lin, Yingbing Ma, Yi Zhao, Yi Liu, Chunyu Tan*


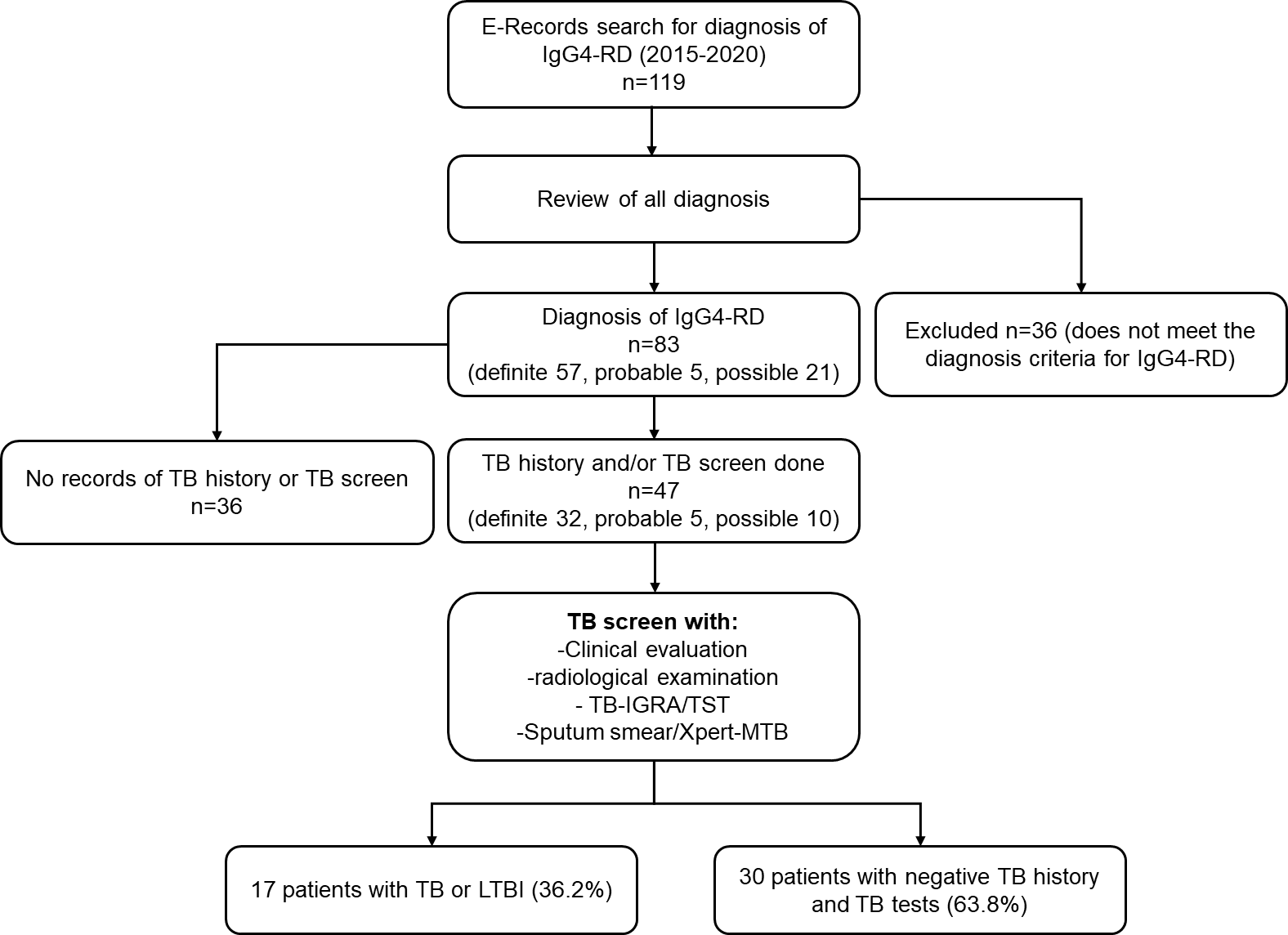


**Figure S1. Flow chart of the retrospective IgG4-RD cohort.** Diagnoses in the electronic charts of patients of the Sichuan University West China Hospital from Feb 2015-Sep 2020 were screened for ‘IgG4-RD’. Within patients with the diagnosis of IgG4-RD, we identified 83 patients meeting the 2011 comprehensive diagnostic criteria for IgG4-RD ([1](#_ENREF_1)), including 57 definite, 5 probable and 21 possible cases. Patients dose not meet the diagnosis criteria were excluded (n=36). Among patients with IgG4-RD, we noted 47 patients were inquired TB history and/or TB screen including TB-IGRA, Tuberculin skin test (TST), Xpert-MTB and sputum smear. Notably, 17 IgG4-RD patients (36.2%) were complicated with TB disease or latent TB infection (LTBI). The other 30 patients were negative for TB history and TB tests (63.8%).

**Reference**

1. Umehara H, Okazaki K, Masaki Y, Kawano M, Yamamoto M, Saeki T, et al. Comprehensive diagnostic criteria for IgG4-related disease (IgG4-RD), 2011. Mod Rheumatol. 2012;22(1):21-30.
